# Metagenomic-based surveillance systems for antibiotic resistance in non-clinical settings

**DOI:** 10.3389/fmicb.2022.1066995

**Published:** 2022-12-02

**Authors:** Stephanie Pillay, David Calderón-Franco, Aysun Urhan, Thomas Abeel

**Affiliations:** ^1^Delft Bioinformatics Lab, Delft University of Technology, Delft, Netherlands; ^2^Department of Biotechnology, Delft University of Technology, Delft, Netherlands; ^3^Infectious Disease and Microbiome Program, Broad Institute of MIT and Harvard, Cambridge, MA, United States

**Keywords:** antibiotic resistance, metagenomics, bioinformatics, microbiomes, gene transfer

## Abstract

The success of antibiotics as a therapeutic agent has led to their ineffectiveness. The continuous use and misuse in clinical and non-clinical areas have led to the emergence and spread of antibiotic-resistant bacteria and its genetic determinants. This is a multi-dimensional problem that has now become a global health crisis. Antibiotic resistance research has primarily focused on the clinical healthcare sectors while overlooking the non-clinical sectors. The increasing antibiotic usage in the environment – including animals, plants, soil, and water – are drivers of antibiotic resistance and function as a transmission route for antibiotic resistant pathogens and is a source for resistance genes. These natural compartments are interconnected with each other and humans, allowing the spread of antibiotic resistance *via* horizontal gene transfer between commensal and pathogenic bacteria. Identifying and understanding genetic exchange within and between natural compartments can provide insight into the transmission, dissemination, and emergence mechanisms. The development of high-throughput DNA sequencing technologies has made antibiotic resistance research more accessible and feasible. In particular, the combination of metagenomics and powerful bioinformatic tools and platforms have facilitated the identification of microbial communities and has allowed access to genomic data by bypassing the need for isolating and culturing microorganisms. This review aimed to reflect on the different sequencing techniques, metagenomic approaches, and bioinformatics tools and pipelines with their respective advantages and limitations for antibiotic resistance research. These approaches can provide insight into resistance mechanisms, the microbial population, emerging pathogens, resistance genes, and their dissemination. This information can influence policies, develop preventative measures and alleviate the burden caused by antibiotic resistance.

## Introduction

Antibiotic-resistant bacterial infections have contributed to 4.95 million deaths worldwide, with 1.27 million of those directly resulting from antimicrobial resistance (AMR; Gigante et al., 2022; Papavarnavas and Mendelson, 2022). It is the leading cause of death worldwide and affects high, low, and middle-income countries (Pokharel et al., 2019; Gupta and Gupta, 2021). Antibiotic-resistant bacterial infections are rising exponentially, making them harder to treat (Dušan et al., 2016; Robinson et al., 2016). This continuation will lead to an estimated 10 million deaths annually by 2050 if the burden of AMR is not alleviated (Van Boeckel et al., 2015).

For most of human history, bacteria have caused infectious diseases and contributed to high mortality rates (MacLean and San, 2019). To counteract this, antibiotics were routinely prescribed to treat and prevent such diseases. This saved millions of lives each year. Due to its success, antibiotics were used in animal husbandry and agricultural practices with the added benefits of growth promotion (Kang et al., 2018). Unfortunately, this success was limited to the emergence of antibiotic-resistant bacteria (ARB) and its effects on human health and longevity (Fair and Tor, 2014; MacLean and San, 2019; Kumar et al., 2020).

The continuous and unwarranted use of antibiotics in humans, animals, and agricultural areas exerts selection pressure on the bacteria found in these sectors. This gives rise to bacteria resistant to multiple antibiotics classes leading to untreatable infections (Davies and Davies, 2010; Cabello and Godfrey, 2016).

The ability of bacteria to resist antibiotics is embedded in their evolutionary history, which has led to various phenotypic and genotypic resistance mechanisms (Pornsukarom and Thakur, 2017; Power, 2019). These include the inactivation, modification, degradation, and expulsion of the antibiotic or its target site, therefore protecting the bacteria (Capita and Alonso-Calleja, 2013; Blair et al., 2015; Munita and Arias, 2016; Kumar, 2017; Kang et al., 2018). It is also possible that bacteria gain resistance through mutations, changing the nature of proteins expressed in the bacterial organism, or by horizontal gene transfer phenomena, which is the exchange of genetic material by the use of mobile genetic elements (MGEs) between bacterial strains (Pornsukarom and Thakur, 2017; MacLean and San, 2019). This allows bacteria to collect numerous resistant traits and ultimately become multi-drug resistant (MDR; Munita and Arias, 2016; von Wintersdorff et al., 2016; Kumar, 2017; Power, 2019).

The ineffectiveness of antibiotics threatens the ability to treat common bacterial infections and minor injuries. Since first-line antibiotics are deemed sub-par, more expensive treatment, alternative therapy and additional care is necessary. This can result in prolonged infections and an increase in morbidity and mortality rates. This puts an economic burden on families, society, and modern medicine (Gandra et al., 2014; Friedman et al., 2016; Aslam et al., 2018; Power, 2019).

For over a decade, antibiotic resistance has been studied using the traditional culture-dependent approach. This involves the use of artificial conditions to culture microorganisms. Unfortunately, some microorganisms cannot be cultured by this approach (Schmieder and Edwards, 2012; Nowrotek et al., 2019). Approximately 80% of bacterial species in the human gut and 99% of environmental bacterial species remain uncultured (Lagier et al., 2012; Nowrotek et al., 2019). Factors such as slow growth, microbial competition, specific growth requirements, and environmental stressors can affect the culturing process. This leaves limited information regarding gene transfer between these bacterial communities (Garmendia et al., 2012; Schmieder and Edwards, 2012; Suenaga, 2012; Nowrotek et al., 2019; Moralez et al., 2021).

Whole metagenome DNA sequencing can describe the genomes of the total microbial community found in nature. This approach enables the study of culturable and non-culturable bacteria by bypassing the need for isolation and laboratory cultivation of microorganisms. DNA directly isolated from the environmental sample can broaden the understanding of the structure, gene/species richness and distribution, and the functional and metabolic potential of a microbial community (Suenaga, 2012).

Applying this approach to antibiotic resistance in different microbial communities can identify known and novel resistance genes and mobile genetic elements, i.e., plasmids, integrons, transposons, and phage’s (Partridge et al., 2018; Stalder et al., 2019; Hall et al., 2022; Yao et al., 2022). This information is the stepping stone to making new policies for infection and prevention control measures, thereby reducing the incidence of infection and optimizing the use of antibiotics by health professionals, in the healthcare, animal, and agricultural industries. In addition, it will provide improved awareness and understanding of antibiotic resistance as a whole.

Unfortunately, AMR monitoring systems focus on clinical and the public health sectors as a representation of AMR as a whole. Organizations such as WHO and the implementation of the Global Antimicrobial Surveillance System (GLASS) have relied on the use of traditional culturable methods for clinical AMR research. This creates a biased outlook on AMR as non-clinical sectors, and non-culturable bacteria are excluded. This review will focus on AMR in non-clinical sectors and the use of bioinformatics as an aid in this research.

## DNA sequencing technologies used in antibiotic resistance research

Microbiomes are complex and require sequencing technologies to be comprehensive enough to capture the present representative sequences from species. Several sequencing platforms can be used to study AMR in clinical and non-clinical sectors. They are useful in detecting antibiotic resistance genes (ARGs), virulence factors, MGEs, and diversity of the microbial community (Forbes et al., 2017). The advancements in metagenomics have been driven by next-generation sequencing technologies (NGS) i.e., second and third-generation sequencing technologies (Table 1).

**Table 1:** Second-generation versus third-generation sequencing technologies for antibiotic susceptibility testing.

	2^nd^ Generation	3^rd^ Generation
Definition	NGS technology where the nucleic acid is fragmented and amplified followed by sequencing of many different clusters of short reads taking place in parallel where base detection is monitored by different platforms such as Illumina	NGS technology allows for long-read single-molecular sequencing by monitoring the incorporation of fluorescently labelled nucleotides or by monitoring an electric single as nucleic acid is fed through a nano-sized pore
Advantages	+ High reproducibility Requires less DNA/RNA (ng) Strain typing Resistance determinants such as single nucleotide polymorphism level analysis Detection of chromosomal mutations leading to AMR Detection of ARGs and allelic variants	+ Suitable for pathogen identification High sensitivity for microorganism detection Detection of ARGs Longer reads allow for the detection and mapping of mobile genetic elements in multi-drug-resistant strains Provides genetic context
Limitations	Results in days therefore cannot be used as a rapid diagnostic tool Complex analysis due to high volumes of data and short reads Unable to link ARGs to genetic context Short reads result in fragmented assemblies around repetitive regions associated with ARGs or mobile genetic elements	High cost Resistance determinants such as single nucleotide polymorphisms cannot be analysed Chromosomal mutations leading to AMR cannot be detected Allelic variants cannot be detected A high concentration of input nucleic acid is required
Platforms	Life Technologies (Ion Torrent and Ion Proton) Illumina (HiSeq, MiSeq, GenomeAnalyser)	Pacific Biosciences (PacBio RS) Oxford Nanopore (MinION, PromethION, GridION)

Second-generation sequencing has been the most widely used sequencing technology for microbial genomics. Second-generation sequencing technologies, i.e., Illumina and Ion Torrent, produce short reads from 100 up to 300 base pairs with high accuracy (~0.1% error rate; De Maio et al., 2019; Yee and Simner, 2019). These short reads provide information on species population, evolutionary relationships, allelic variations, SNPs, and the detection of specific genes, i.e., ARGs (Boolchandani et al., 2019; De Maio et al., 2019; Yee and Simner, 2019). Although short reads provide information on the genomic content of the bacterial isolates in a sample, it does not provide information on the genomic structure, i.e., which genes are present on chromosomes and MGEs such as plasmids which are crucial in understanding the dissemination of ARGs (George et al., 2017; De Maio et al., 2019; Yee and Simner, 2019; Maguire et al., 2020).

Third-generation technologies overcome this limitation by providing a better view of the genetic structure as longer reads (on average 1–100 Kbp) span most of the repetitive sequences, linking ARGs to MGEs, e.g., plasmids (Boolchandani et al., 2019; Yee and Simner, 2019). This long-read technology is advantageous for studying AMR in bacterial isolates, and metagenomic samples as the complexity of assembly is reduced (De Maio et al., 2019). Unfortunately, the high error rate makes it difficult to identify specific allelic variants or SNPs in chromosomal genes leading to AMR (Yee and Simner, 2019). On the other hand, Pacbio and ONT are easily accessible and able to produce raw data in real-time, making them a good tool for the rapid diagnostics (Che et al., 2019). Long reads are also a practical method to determine the species/gene richness, distribution, and functional potential of a microbial community (Garmendia et al., 2012; Crofts et al., 2017).

While third-generation technologies are successful in AMR research, it is limited by the high error rate and low accuracy (Yee and Simner, 2019). To overcome this, a combination of short-reads (i.e., Illumina) with long-reads (i.e., ONT) is a promising way to generate fully resolved and accurate bacterial genome assemblies which characterize antibiotic resistance genes on plasmids and genomes from environmental samples (Liu et al., 2020; Chen et al., 2021; Moralez et al., 2021). This combination allows for better assembly of complex genomes as long reads provide information on the structure of the genome, and short reads can be used to correct errors in long reads (De Maio et al., 2019).

These sequencing technologies directly characterize the microbiomes in humans, animals, and environmental samples, providing high-throughput sequencing of either whole genomes, targeted amplicons, or whole metagenomes (Di Bella et al., 2013).

Whole-genome sequencing (WGS) uses DNA sequencing technologies to sequence the entire genome of isolated pure organisms to characterize genomic variants (Table 2; Collineau et al., 2019). WGS overcomes the limitations of traditional culture-dependent approaches and phenotypic tests, i.e., disk diffusion for AMR (Balloux et al., 2018). By opting to use WGS, more information on the bacterial genetic determinants conveying resistance and their association with mobile genetic elements can be established (Balloux et al., 2018; Oniciuc et al., 2018). This contributes to outbreak detection, infection control, and epidemiological surveillance, all of which are an essential part of the AMR surveillance (Collineau et al., 2019).

**Table 2:** Metagenomic sequencing vs. other techniques used to study qualitatively and quantitatively antibiotic resistance.

	Whole-genome sequencing	Meta-proteomics	Whole-metagenome sequencing	Quantitative PCR
Definition	The sequencing of an entire genome of a single organism is obtained from a culture to identify species/ strains, genes, and mutations associated with AMR.	Identification and quantification of proteins conferring AMR and Multi-drug resistance (MDR) to localized MGEs from microbial communities.	A culture-independent approach is used for the identification of all ARGs/ mutations in all organisms present in complex microbial communities.	Method for the detection, quantification, and typing of specific microbial species/ strains or ARGs/ markers.
Technique	DNA is extracted from bacterial culture and sequenced to generate FastQ reads.	The generation of peptides and high-resolution mass spectra analysis to identify and quantify proteins.	DNA is extracted directly from a sample and sequenced to generate reads representing the entire microbial community.	An enzymatic reaction in combination with fluorophores (SYBR Green and TaqMan) is used to amplify a specific target of interest and quantify gene expression levels.
Advantages	+ Reproducible High resolution Standardization of analysis methods Rapid turn-around time High specificity Read lengths of 1 – 50Kb	Accurate prediction Large-scale protein identification Rapid turn-around time	+ High resolution Accurate prediction	Fast High-throughput detection and quantification of target DNA sequences avoid cross-contamination Sensitivity Specificity
Limitations	- Requires cultured organism Computationally demanding Cost	- False positives from large datasets False discovery rates in large datasets Limited reproducibility	- Difficult to differentiate between host and pathogen Expensive Computationally demanding	- Incapable of distinguishing between live and dead cells
Outcome	Microbial typing and tracing Ability to predict AMR from only genomic data Discovery of novel resistance genes or mutations	Host-pathogen interaction Virulence factors Antibiotic resistance mechanisms Identification of functions involved in biological pathways	Species-or strain level identification AMR Virulence potential Detect Mobile genetic elements Discovery of viruses	Determine the presence of specific genes and alleles AMR profiling Toxin production Typing of strains and isolates

Amplicon sequencing uses marker genes such as 16S (prokaryotes) or 18S rRNA (eukaryotes) genes. Both approaches require the DNA extracted directly from the microbial community and are subjected to either direct sequencing or amplification *via* polymerase chain reaction (PCR; Table 2). In both instances, the sequences are queried against a reference database (Table 3). This is a reliable process to identify the composition of a microbial community down genus-level within a sample (Ranjan et al., 2016; Forbes et al., 2017; Oniciuc et al., 2018).

**Table 3:** General and specific databases in AMR research.

General databases	Description	Update	Website	Reference
ResFinder and ResFinderFG	A curated database that identifies acquired genes and/ or finds chromosomal mutations.	Apr-21	https://cge.cbs.dtu.dk//services/ResFinder/	(Zankari et al., 2012; Xie et al., 2018)
CARD	Manually curated database and an ontology resource that provides information on ARGs and resistance mechanisms.	Jul-21	https://card.mcmaster.ca/	(Ahmed et al., 2007; Jia et al., 2016; Chen et al., 2017; Guo et al., 2017)
SARG (v2)	ARG database derived from ARDB, CARD, and NCBI-NR databases for characterization and quantification of ARGs.	Apr-21	http://smile.hku.hk/SARGs	(Yin et al., 2018)
FARME	Curated database focused on environmentally derived metagenomes conferring resistance.	Mar-19	http://staff.washington.edu/jwallace/farme/	(Wallace et al., 2017)
MEGARES	Database containing sequence data for approximately 8,000 manually curated ARGs.	Oct-19	https://megares.meglab.org/	(Lakin et al., 2017)
MUSTARD	Database of 6,059 AMR determinants from 20 families in the human gut microbiota and curated sets of genes. Identified *via* functional metagenomics.	Sep-17	http://mgps.eu/Mustard/	(Ruppé et al., 2019)
RESFAMS	A profile HMM-based curated database to confirm antibiotic resistance function and organized by ontology.	Jan-15	http://www.dantaslab.org/resfams/	(Gibson et al., 2015)
NDARO	Curated and collated data from multiple databases. Contains 5,804 sequences.		https://www.ncbi.nlm.nih.gov/bioproject/PRJNA313047	
Specific Databases	Description	Update	Website	Reference
LacED	Curated database providing information on mutation, sequences, and structures of TEM and SHV β-lactamases.	TEMLacED: 2017 SHVED:2010	http://www.laced.uni-stuttgart.de/	(Thai et al., 2009)
CBMAR	Database identifying and characterizing novel β-lactamases based on Amber classification.	Sep-14	http://proteininformatics.org/mkumar/lactamasedb/	(Srivastava et al., 2014)
BLDB	A manually curated database of AR enzymes classified by class, family, and subfamily	Jul-21	http://bldb.eu/	(Naas et al., 2017)

Metagenomic sequencing involves the fragmentation, sequencing, assembly, and annotation of the total genomic DNA isolated in a given sample (Oniciuc et al., 2018; Xu et al., 2021). Similar to WGS, whole metagenome sequencing (WMS) can provide information on the entire gene content of either prokaryotic and eukaryotic organisms as well as species or strain-level identification and virulence or resistance potential (Ranjan et al., 2016; Quainoo et al., 2017; Hendriksen et al., 2019). In addition, WMS makes it possible to predict the metabolic potential of the microbial community. This comprehensive view of all gene content allows access to information on AMR that is essential for setting up control and prevention strategies in all sectors (Xu et al., 2021).

Meta-proteomics focuses on functional change in a microbiome. Assisting in the control strategies and plays a key role in identifying molecular mechanisms of bacterial pathogenesis and disease outcome determinants. It can aid in developing pathogen-specific treatment strategies that can lower the spread of AMR. Meta-proteomics is reliable for reviewing bacteria in soil, sludge, food, and the ocean (Wilmes and Bond, 2004; Bastida et al., 2014; Williams and Cavicchioli, 2014; Maier et al., 2017). Unfortunately, there are inconsistent protocols for sample preparation, inefficient bioinformatic tools, and challenges in measuring low-abundance proteins within a complex protein sample (Zhang et al., 2018). This technique, while reliable, should be applied with other sequencing technologies to have an in-depth analysis of microbial communities, antibiotic resistance genes, and host-pathogen interaction (Zhang and Figeys, 2019; Khodadadi et al., 2020).

## Bioinformatic analysis of metagenomic sequences for antibiotic resistance research

The constant development of new computational pipelines is advantageous in providing an accurate depiction of the resistome from metagenomic sequencing data. These analyses rely on a variety of algorithms: quality control of DNA sequencing data, genome and metagenome assembly algorithms, read-mapping, variant detection, phylogenetics, taxonomic databases, and visualization (McArthur and Tsang, 2017). AMR databases are discussed in more detail in the next section.

**Figure 1:**
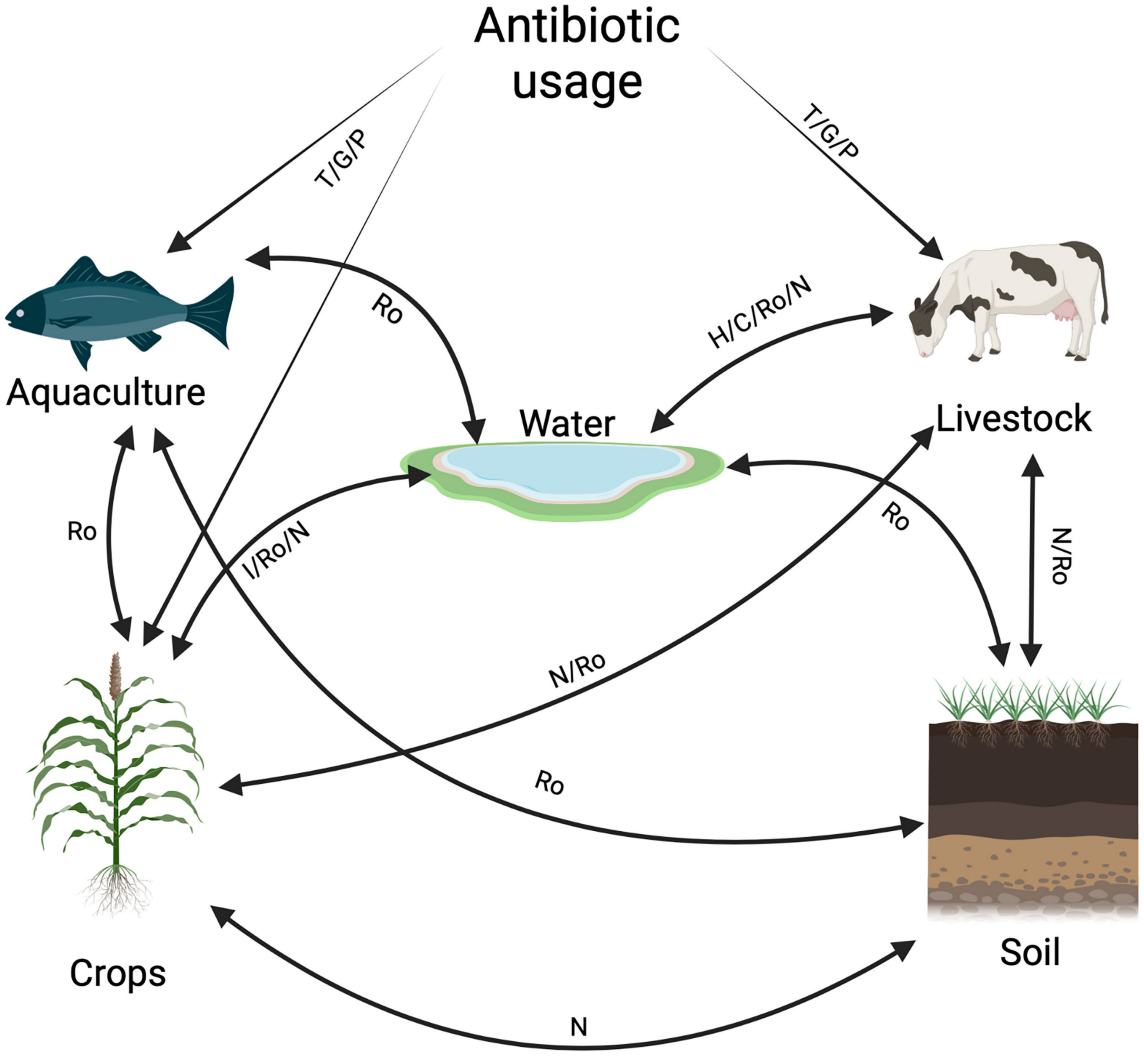
Pathway map of antibiotic agents and antibiotic resistance dissemination between livestock, agriculture, aquaculture, and water. The movement of antibiotic resistance or antibiotic agents is indicated by solid lines. The potential routes of transmission and exposure between non-clinical sectors and antibiotic usage is indicated by the abbreviations: Ro: run-off /leaching, T: treatment, P: prevention, GP: growth promoter, H: animal house, C: cleaning, N: nutrients, I: irrigation (Created with BioRender.com). Routes between non-clinical sectors (↔) and antibiotic usage in non-clinical sectors (→) have been described by the previous studies mentioned. Water ↔ crop: (Hu et al., 2010; Gatica and Cytryn, 2013; Meena et al., 2015; Bürgmann et al., 2018; Rodríguez-Molina et al., 2019), water ↔ soil: (Hu et al., 2010; Seiler and Berendonk, 2012; Michael et al., 2013; Meena et al., 2015; Tang, 2016), water ↔ livestock: (Miyata et al., 2011; Hao et al., 2014; Van Boeckel et al., 2015; Magouras et al., 2017; Madec and Haenni, 2018; McEwen and Collignon, 2018; Opatowski et al., 2021), water ↔ aquaculture: (Seiler and Berendonk, 2012; Shah et al., 2014; Grenni et al., 2018; Pérez-Sánchez et al., 2018; Yuan et al., 2019; Preena et al., 2020a,b; Reverter et al., 2020; Sonone et al., 2020), antibiotic use → aquaculture: (Shah et al., 2014; Pérez-Sánchez et al., 2018; Stärk et al., 2019; Preena et al., 2020b), antibiotic use → livestock: (Ahmed et al., 2007; Marshall and Levy, 2011; Landers et al., 2012; Hao et al., 2014; Speksnijder et al., 2015; Van Boeckel et al., 2017; McEwen and Collignon, 2018), antibiotic use → crops: (Hirniesen et al., 2012; Pan and Chu, 2017; Sundin and Wang, 2018; Zhu et al., 2019; Gao, 2021), livestock ↔ soil and crops: (Popowska et al., 2012; Wang et al., 2015; Lin et al., 2016; Chen et al., 2017; Xie et al., 2018; Pérez-Valera et al., 2019; Zhang et al., 2019).

The typical process to identify antibiotic resistance genes from sequenced metagenomes is based on one of two general techniques: (i) read-mapping and (ii) assembly. Each of these could be followed by binning or an annotation step (Table 4). A bioinformatics pipeline for analysing metagenomic data for antibiotic resistance research has been presented in Figure 1.

**Table 4:** Various bioinformatics approaches for the prediction of AMR and MGEs.

	Read-mapping approach	Assembly-based approach	Binning approach	Annotation
Definition	The detection of genes without genome assembly. The direct alignment of reads to a reference database.	The identification of genes with *de novo* assembly profiling.	The evaluation of taxonomic groups by clustering assembled sequences into individual groups that represent microbial species.	The prediction of genes from either assembled or unassembled reads.
Function	Antibiotic resistance gene discovery and taxonomic identification.	Antibiotic-resistant gene discovery and taxonomic identification.	Assessment of taxonomic diversity and gene association to taxonomic groups.	Gene function and taxonomic identification.
Technique	High-quality reads are directly aligned to a reference database using pairwise alignment tools or splitting reads into k-mers and mapping to reference databases.	High-quality reads are assembled into contigs that are aligned to a reference database using BLAST (Altschul et al., 1990) or HMMscan.	The process of clustering contigs into individual genome bins by machine learning methods or comparing metagenomic sequences against a reference database of genomic sequences.	Sequences are classified by coding and non-coding regions to determine gene function.
Advantages	+ Fast Less computationally demanding Identify genes from low abundance organisms in complex communities Resistome analysis of large datasets	+ Identification of protein-coding regions Identification of known and novel resistance genes Regulatory and mobile genetic element sequences identified	+ Easy Reliable	+ High accuracy High sensitivity
Limitations	- Lacks information required to study upstream and downstream factors of identified resistance genes Large datasets may cause false-positive predictions	- Loss of information due to incomplete and poor assembly Computationally expensive Time-consuming Requires a high genome coverage	- Lack of whole-genome sequences within the available databases	Computationally demanding Limited success for short reads Expensive
Tools	SRST2 (Inouye et al., 2014), ARIBA (Hunt et al., 2017), BOWTIE2 (Langmead and Salzberg, 2012), Burrows-wheeler aligner (BWA) (Li and Durbin, 2009)	MEGAHIT (Li et al., 2015), MetaSpades (Nurk et al., 2017), MetaVelvet (Namiki et al., 2012), Spades (Bankevich et al., 2012), Velvet, Abyss (Simpson et al., 2009) and SOAPdenovo (Luo et al., 2012)	MetaBat2 (Kang et al., 2019), Maxbin2 (Wu et al., 2016), CONCOCT (Alneberg et al., 2014), GroopM (Imelfort et al., 2014), DASTool (Sieber et al., 2018). MetaCluster (Leung et al., 2011), MEGAN (Huson et al., 2007), MGRAST (Glass et al., 2010)	RAST (Aziz et al., 2008), IMG (Markowitz et al., 2009), MetaGeneAnnotator (Noguchi et al., 2008), MetaGeneMark (McHardy et al., 2007)

**Figure 2:**
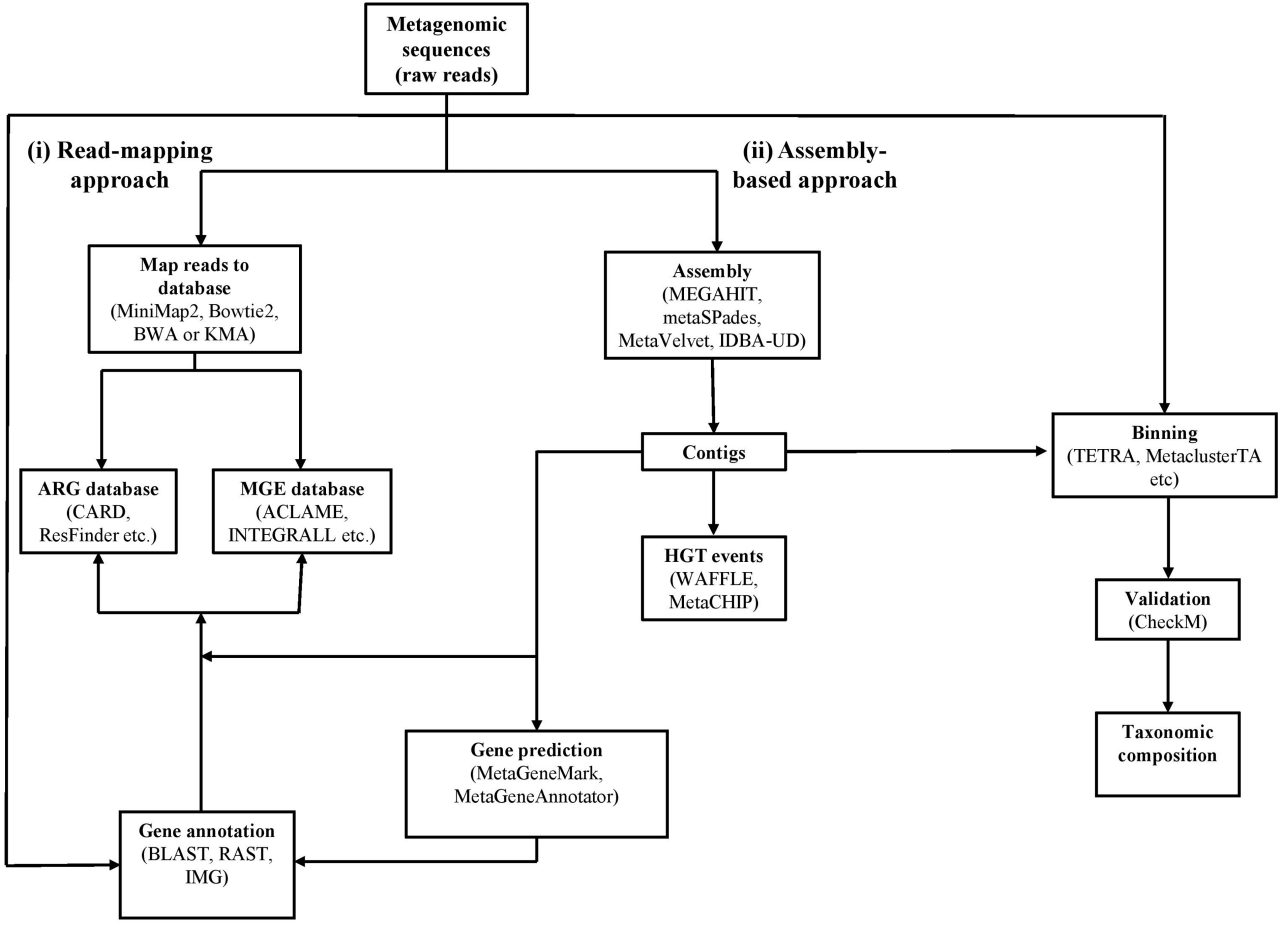
Workflow for determining antibiotic resistance genes, mobile genetic elements, horizontal gene transfer events and taxonomic composition using metagenomic data. The workflow is indicated by solid lines with example tools and databases annotated in the boxes. The complete lists are described in Tables 3, 4.

Read mapping allows for raw read sequences to be directly aligned to reference databases (Table 3) using pairwise tools such as MiniMap2 (Li, 2018), Bowtie2 (Langmead and Salzberg, 2012), Burrows-wheeler aligner (BWA; Langmead and Salzberg, 2012), or by splitting reads into k-mers and mapping to a reference database using k-mer alignment (KMA; Clausen et al., 2016; Figure 2). This provides information on the similarity of one sequence to another by counting the number of alignments to quantify the abundance of similar sequence patterns (Clausen et al., 2016).

In the last decade, read-mapping approaches were deemed superior to assembly approaches for AMR gene detection, i.e., reads are first assembled and mapped to a database using BLAST (Johnson et al., 2008; Hunt et al., 2017; Clausen et al., 2018). The success of an assembly-based approach is highly dependent on the quality of the assembly (Inouye et al., 2014; Clausen et al., 2016). An approach as such is problematic as genes of interest, i.e., ARG, s can be split over two or more contigs and will not be identified if the assembly is poor quality (Clausen et al., 2016, 2018).

Read-mapping based methods overcome this by using the tools above, which allow for fast mapping and alignment of raw reads against large reference genomes and entire databases (Clausen et al., 2016). This can identify genes from low abundance organisms in a complex community with speed and ease of computation. Since epidemiological databases are constantly being updated with new sequences due to natural evolution, the read-mapping approach becomes difficult as results constantly change. There is no guarantee that the read will cover a unique part of the reference sequence and will result in a tie for the best match due to the random selection (Clausen et al., 2016). Fortunately, tools like SRST2 (Inouye et al., 2014) resolve the ties by pre-and post-processing of sequences for read-mapping, which can predict the presence of ARGs in a sample (Clausen et al., 2018; Taş et al., 2021).

Identification and characterization of ARGs can also be achieved using assembly-based approaches (Table 4; Figure 2; Breitwieser et al., 2019). This method allows reads to be assembled into contigs and then queried against reference databases (Table 3). The assembly of WMS data is complicated as there is an uneven or unknown abundance of different genomes (Ghurye et al., 2016).

Unrelated genomes may also contain nearly identical DNA repeats, which could represent MGEs (Boolchandani et al., 2019). In addition, multiple individual organisms could be from the same species but may harbour small genetic differences indicating strain variants (Ghurye et al., 2016). Technical factors, e.g., library preparation, sequencing depth, and sequencing platforms, affect the accuracy in assembling WMS data into larger contigs (Escobar-Zepeda et al., 2015). Metagenomic assemblers such as MEGAHIT (Li et al., 2015), MetaSPades (Nurk et al., 2017), IDBA-UD (Peng et al., 2012), and MetaVelvet (Namiki et al., 2012) have been developed to overcome these challenges by normalizing sequencing depth, correcting read errors, and detecting and reporting genomic variants and repeats (Ghurye et al., 2016; Vosloo et al., 2021). To date, no single assembler is the best at accurately reconstructing known genomes and capturing taxonomic diversity in a metagenomic sample. Multiple assemblers are recommended to be applied to a subset of samples to determine which has the best fit (Escobar-Zepeda et al., 2015; Ghurye et al., 2016; Boolchandani et al., 2019).

Binning classification can predict the taxonomical composition using information contained in reads (Figure 2). Reads are grouped to represent an individual genome or closely related genomes (Table 4). Binning can be done either with reads or assembled sequences and employs two strategies to obtain taxonomic assignment; (i) sequence composition classification and (ii) sequence alignment against references. The first is based on looking at genomic signatures using k-mers to identify evolutionary conservation among species and uses software such as TETRA (Teeling et al., 2004), MetaClusterTA (Wang et al., 2014), and PhylophytiaS (McHardy et al., 2007). Similarly, software like MaxBin (Wu et al., 2016) and Amphora2 (Wu and Scott, 2012) use k-mer signatures but also considers gene markers, GC content, and coverage information for binning on assembled sequences or reads. Another method based on reference read alignment is the Burrows-Wheeler Transform (Burrows and Wheeler, 1994) which indexes like BWA (Li and Durbin, 2009) or Bowtie (Langmead and Salzberg, 2012) and is fast and accurate in assessing species richness and abundance in WMS by aligning them to reference genomes. Genometa (Davenport and Tümmler, 2013) is a software that looks at OTUs in the WMS data and groups according to genomic islands and operons. Taxonomic classification in long reads using the potential coding regions to search in annotated protein databases using BLAST (Johnson et al., 2008) or Megan (Huson et al., 2007). Binning achieved after the assembly of contigs can lead to the generation of partial genomes of unknown or uncultured organisms. This can be used to perform similarity-based binning of other metagenomic datasets. If binning is achieved before assembly, it can reduce the complexity of an assembly-based approach and may reduce the computational demands. However, caution should be taken to ensure the validity of the genome bins as there is a chance of false assignments (Thomas et al., 2012; Bengtsson-Palme, 2018a). In this instance, CheckM (Parks et al., 2015) can be used to evaluate the quality of bins on metagenomic samples and assess the quality of assembled metagenomic genomes by estimation of completeness and contamination based on marker-gene validation (Du and Sun, 2022).

Taxonomic assignments of reads can also be done with genome annotation. This process identifies the coding regions and their location to determine gene function. Annotation of metagenomic sequences can be done in two ways, (i) existing pipelines, e.g., RAST (Aziz et al., 2008) and IMG (Markowitz et al., 2009), can be used for assembled genomes, and (ii) annotation can be performed on an entire community and relies on unassembled reads and short contigs. WMS data is annotated by identifying genes (feature prediction) and assigning putative gene functions and taxonomic neighbours (functional annotation; Thomas et al., 2012). Prediction tools such as MetaGeneAnnotator (Noguchi et al., 2008) or MetaGeneMark use internal information to classify sequences as either coding or non-coding and are done with a low error rate of 2% (Thomas et al., 2012).

## Antibiotic resistance databases

The performance of antibiotic resistance gene prediction and taxonomic identification depends on the availability of accurate databases. Two types of public databases exist, generalized AMR databases, which include a wide range of ARGs and mechanistic information, and specialized databases, which provide extensive information on specific gene families, e.g., the β-lactamase family (Hendriksen et al., 2019).

Several bioinformatic tools and databases can be used in AMR research. They focus on fast and reliable predictions of ARB and their genetic determinants in complex communities (Van Camp et al., 2020). It is important to note that the quality and type of sequence play an essential role in AMR research. The bioinformatics tool’s robustness can differ when analysing sequences of low-quality (Hendriksen et al., 2019). Any database’s value depends on maintenance, curation, and continuous updates with detailed metadata. Correctly predicting ARGs from large-scale datasets with improved accuracy depends on input parameters such as e-values, bit scores, identity, and query coverage levels (Lal Gupta et al., 2020).

### General antibiotic resistance databases

General antibiotic resistance databases incorporate a wide variety of ARGs and mechanistic information.

Established tools like ResFinder (Zankari et al., 2012) accept short reads and contigs to detect the presence of acquired ARGs. This can be done for complete or partial genome sequences and uses BLAST (Johnson et al., 2008), or KMA (Clausen et al., 2018) approaches (Zankari et al., 2012; Table 3). The specificity of using BLAST to detect antibiotic resistance genes depends on the selection criteria for gene length and percentage similarity. This method requires computational expertise and could be challenging to identify multiple copies of ARGs in *de novo* assembled genomes (Clausen et al., 2018; Hameed and Fatima, 2021). This can be solved by using longer reads which increases the cost.

In comparison, the KMA approach was developed to map the raw reads against redundant AMR databases as the traditional BLAST method remains too slow to map raw reads directly (Clausen et al., 2018; Hendriksen et al., 2019). This approach provides accurate bacterial genome analysis and can identify ARGs present in low abundance, which might be excluded in incomplete assemblies. There is also a chance of false positives due to sequencing errors which can be overcome by setting the minimum threshold for the number of reads needed for a positive outcome (Hendriksen et al., 2019; Hameed and Fatima, 2021).

The Comprehensive Antibiotic Resistance Database (CARD; Jia et al., 2016) for the identification of resistance genes, their products, and associated phenotypes. This database allows for the identification of chromosomal mutations that confer resistance to antibiotics. Similar to ResFinder (Zankari et al., 2012), the percentage coverage and identity can be adjusted to allow for the selection of new ARGs. The Resistance Gene Identifier (RGI), a tool for *de novo* annotation of genes, complete genomes, or genome assembled sequences for AMR, uses CARD’s curated database to predict ARGs in either DNA or amino acid format. This is because some sequences cannot be mapped due to divergence in the nucleotide sequences; however, amino acid sequences in this instance can be translated to proteins to characterize the resistome (Xavier et al., 2016; Lakin et al., 2019). CARD uses two prediction models: a protein homolog model based on functional resistance homologs and a protein variant model that detects mutations conferring resistance (Jia et al., 2016; Imchen et al., 2020). The application of ontologies such as CARD’s Antibiotic Resistance Ontology (ARO) has organized AMR information based on molecular determinants, resistance mechanisms, individual antimicrobials, antimicrobial targets, and drug classes, therefore, making this database favourable (McArthur and Tsang, 2017).

Other curated databases include; MEGARes (Lakin et al., 2017), ResFams (Gibson et al., 2015), NDARO (Sayers et al., 2021), FARME (Wallace et al., 2017), and MUSTARD (Ruppé et al., 2019), all of which are curated for the detection of AMR determinants in large metagenomic datasets (Arango Argoty, 2019; Arango-Argoty et al., 2020; Imchen et al., 2020; Lal Gupta et al., 2020). Interestingly, FARME, a functional antibiotic resistance metagenomic element database, focuses on AMR gene elements from environmental samples (soil, faecal matter, wastewater treatment plants, oral and aquatic biomes) rather than individual ARGs obtained from cultured clinical isolates, providing more access and analysis of non-clinical sectors. This database is compiled from publicly available DNA sequences from 30 functional metagenomic projects and their corresponding predicted proteins conferring AMR (Wallace et al., 2017). FARME contains over seven times the number of non-redundant protein sequences as compared to CARD (Wallace et al., 2017; Lal Gupta et al., 2020). It provides information on regulatory elements, MGEs, and predicted proteins flanking ARGs, which are conserved between functional metagenomic AMR sequences from soil biomes and pathogenic clinical isolates (Forsberg et al., 2012; Wallace et al., 2017). This allows for better insight into AMR in unculturable bacteria found in non-clinical settings (Wallace et al., 2017; Bengtsson-Palme, 2018b; Imchen et al., 2020).

ResFams is a curated database of protein families linked to their profile Hidden Markov Models (HMMs) associated with AMR function are trained using unique AMR protein sequences obtained from CARD, LaCED, and Lahey databases (Lal Gupta et al., 2020). This platform primarily focuses on pathogen-associated ARGs and provides a comprehensive view of resistomes in the environment and the evolution of resistant pathogens (Lal Gupta et al., 2020). ResFams were evaluated with functional metagenomic datasets and demonstrated improved sensitivity, and identified 64% more ARGs in soil and human gut microbiomes compared to BLAST-based searches of CARD and ARDB databases. This increased sensitivity is due to the HMM-based analysis. HMM, models are specific models constructed based on observed sequence variation across genes/protein families and capture possible variation for the families (Xavier et al., 2016). Although this approach is sensitive and can detect distant matches that BLAST-based approaches cannot, ResFams is computationally expensive and requires the user to provide local computational resources to run HMM-based searches (Xavier et al., 2016; Lakin et al., 2019).

### Specific antibiotic resistance databases

Specific databases are created to meet specific needs and provide extensive information about a specific gene family. So far, only specialized databases are available for β-lactamase.

The β-lactamase database (BLAD) is a catalogue of resistance patterns from all classes of β-lactamases collected from NCBI and published data with crystal structures of proteins from the Protein Data Bank (PDB; Berman et al., 2000). Metagenomic sequences can be queried against BLAD to obtain basic information about β-lactamases, search for the gene of interest, identify resistance patterns and search and analyse the 3D structure of the β-lactamase (Danishuddin et al., 2013; Naas et al., 2017).

The CBMAR β-lactamase database (Srivastava et al., 2014) includes detailed biochemical and molecular information to understand known and unknown β-lactamase genes. This provides a cache of information on nucleotides, 3D protein structures, sequence alignments, and mutation profiles.

Similarly, LacED (Thai et al., 2009) is a specialized platform that contains mutational and structural data for TEM and SHV β-lactamases. This is extremely important to understand the evolution of multidrug-resistant pathogens as β-lactamase evolution, and the emergence of new enzymes affects the now available treatment for infections. Furthermore, these resistant genes are present on plasmids which help their spread to different biomes, challenging the treatment options in both clinical and non-clinical sectors (Dandachi et al., 2019).

## Antibiotic resistance in non-clinical sectors

AMR is a natural phenomenon that predates the use of antibiotics. A wide array of novel and clinically characterized ARGs have been detected in environmental samples ranging from pristine environments to agricultural soil (Yang et al., 2019). The major driving force of AMR is the mis-use of antibiotics while other factors such as poor infrastructure, i.e., hygiene and sanitation play a role in maintaining AMR (Collignon et al., 2018; Essack and Sartorius, 2018). Approximately more than 60% of total antibiotic use occurs outside the field of human medicine (Prestinaci et al., 2015; Kumar et al., 2020; Polianciuc et al., 2020). The main non-clinical sectors that are involved in the development of AMR are animal production (food-animals and aquaculture), agriculture (plants), and the environmental (water and soil) compartment. These sectors are interconnected and facilitate the spread of pathogenic ARB within and between them and ultimately to humans. Table 5 summarizes the findings done on AMR in various non-clinical reservoirs through the use of metagenomics.

**Table 5:** Studies focusing on the introduction of antibiotics and AMR in each non-clinical sector.

Reservoirs	Introduction of antibiotics	Study	References
Soil	Organic Manure/agricultural run-off	Identification of unknown tetracycline and sulphonamide resistance genes in forest and grassland soil.	(Willms et al., 2019)
	Manure application	Non-manure amended soil displayed a larger proportion of antibiotic resistance bacteria but carried fewer genes.	(Popowska et al., 2012)
	Manure application /organic compost	Antibiotic resistance genes from manure amended soil accounted for 70% of total resistance genes.	(Su et al., 2014)
	Antibiotic-producing soil bacteria	Thirteen antibiotic resistance genes and two bifunctional proteins conferring resistance to ceftazidime and β-lactamase.	(Donato et al., 2010)
	Antibiotic-producing soil bacteria	Total of eleven ARGs in soil with little human influence.	(Torres-Cortés et al., 2011)
	Fertilizer/ manure application	Tetracycline-resistant bacteria in fertilized soil were three times higher than manure amended soil.	(Kang et al., 2018)
	A fertilizer with heavy metals	ARGs could potentially increase by the use of fertilizer containing heavy metals.	(Lin et al., 2016)
	Manure application	Manure-borne bacteria contribute to the increase of ARGs whereas indigenous soil bacteria prevent the dissemination of ARGs from manure to soil.	(Chen et al., 2017)
	Pollution from a petrochemical plant	The soil was more abundant in ARGs than less contaminated soil.	(Chen et al., 2017)
Plants	Manure application	ARGs on lettuce and endive were grown in manure amended soil. ARGs were detected in the endophytes and phyllosphere of the plants.	(Wang et al., 2015)
	Conventionally produced (manure application) / organic fertilizers	A higher abundance of diverse ARGs and MDR genes were present in the endophyte and phyllosphere of lettuce grown organically.	(Zhu et al., 2017)
	Manure application /fertilizer	More than 50 unique ARGs were detected in the phyllosphere of maize after manure application.	(Chen et al., 2018)
	Compost/ improper sanitation/ run-off from farms	2015 to 2020, five outbreaks linked to green leafy vegetables, two linked to sprouts, and one linked a chopped salad kit in the USA.	(CDC, 2021)
Food-animals	Animal feed / infection prevention	Tetracycline resistant *E. coli* strain present in the gut of chickens receiving food supplemented with tetracycline.	(Marshall and Levy, 2011)
	Animal feed/growth promoter / indirect and direct contact	ARBs were found in each processing step of the beef production chain.	(Yang et al., 2016)
	Animal feed	A total of 495 bacterial species, 50 ARGs were detected in the organic animal feed.	(Eckstrom et al., 2019)
	Therapeutic and non-therapeutic	Antibiotics used in food animals leave antibiotic residues in eggs, milk, and meat products.	(Oliveira et al., 2020)
	Animal feed	Faecal samples from large-scale swine farms showed a total of 146 ARGs.	(Zhao et al., 2018)
	Animal feed	Taxonomic composition analysis showed a decrease of *Proteobacteria* and an increase of *Actinobacteria* in the gut microbiome of male broilers.	(She et al., 2018)
Water	Effluent discharge	The abundance of pathogens and ARGs increased in the effluent after wastewater treatment.	(Tang et al., 2016)
	Chlorination	ARGs are found in high amounts in drinking water suggesting chlorination concentrates ARGs.	(Shi et al., 2013)
	Run-off / discharge	Effluent from municipal wastewater treatment plants and pharmaceutical manufacturing plants releases antibiotics into natural water bodies.	(Guo et al., 2017)
	Reuse of water / potable water	An increase in ARGs in reclaimed potable water samples and associations between 193 ARGs and plasmid-associated genes.	(Garner et al., 2018)
	Sewage run-off	The distinct taxonomic composition of bacterial species found in sewage and the sea suggests sewage is diluted in environmental freshwater.	(Fresia et al., 2019)
Aqua-culture	Animal feed	Fifty-one different ARGs conferring resistance against 24 different antibiotic types with MDR genes located on plasmid sequences.	(Tyagi et al., 2019)
	Animal feed /therapeutic	Tetracycline resistance genes were found in 81% of samples taken from a Chilean salmon farm.	(Shah et al., 2014)
	Animal feed/ therapeutic	An abundance of tetracycline, sulphonamide, and beta-lactam resistance genes which strongly correlated to silver and mercury resistance genes were found indicating co-resistance and co-regulation.	(Yuan et al., 2019)

### Food-animals

Each year, half of the antibiotics produced are used for industrial farm animal production (Schmieder and Edwards, 2012). The global demand for animal protein has directly driven the use of antibiotics in livestock. The food-animal industry is a hotspot for antibiotic usage, estimated to increase by 67% in highly populated countries (Van Boeckel et al., 2015). Antibiotics are administered as metaphylactic and prophylactic treatment for the whole flock or herd to prevent the spread of infectious diseases. Furthermore, it is used as a growth-promoting agent across the globe (Van Boeckel et al., 2015; Founou et al., 2016; Robinson et al., 2016).

Historically, the negative effects of antibiotic usage in livestock production have been overlooked because of the drive to keep up with consumer demand (Robinson et al., 2016; Ramtahal et al., 2022). This has led to the prolonged use and dosage, creating an ideal condition for the emergence of ARB and ARGs (Marshall and Levy, 2011; Madec and Haenni, 2018).

AMR can spread through direct and indirect contact, which was confirmed by finding the same ARBs in the poultry farm and the caretaker (Wee et al., 2020). Antibiotics and their residues can be found in most connecting areas such as soil and plants, and animal products which can lead to health risks when consumed due to the direct toxicity, allergic reactions, carcinogenic effects, and disturbance of the beneficial microbiota in children, those who are pregnant, the elderly, and people living with immunocompromised people (Oliveira et al., 2020).

Areas such as the EU and United States have made efforts to ban the use of antibiotics as growth enhancers. However, they are still widely used in South Africa, Brazil, India, China, and Russia (Van Boeckel et al., 2015; Robinson et al., 2016). This strategy is based on the assumption that susceptible strains can outnumber AMR microorganisms if the advantage of becoming resistant is decreased (Andersson and Hughes, 2011). This was supported by a post-ban study conducted in Denmark and Norway. Interestingly, even though ARB decreased, the broilers that were studied were still colonized by ARB years after the ban came into effect. It is such that the ban on antibiotics is still up for debate as decreasing antibiotic use could increase the frequency of bacterial infections (Holmes et al., 2016; Andersson and Hughes, 2019).

The World Health Organization has recommended that farmers and the food industry stop using antibiotics for the growth and prevention of disease in healthy animals. Farms should consider the employment of proper preventive measures and farming practices to limit the risk of bacterial infections instead of the routine antibiotic dosing of animals to prevent disease. If this is done correctly, we could see the decreased use of antibiotics and the alleviation of (Marshall and Levy, 2011; Robinson et al., 2016; Aslam et al., 2018; Van Gompel et al., 2020).

### Water

Water represents one of the most important bacterial habitats and is a major pathway and reservoir for disseminating microorganisms and AMR. This is due to the spread of AMR by humans, and animals, or contamination of the environment by pathogens (Hutton and Chase, 2017; Miłobedzka et al., 2021).

#### Grey water

Wastewater treatment plants (WWTPs) collect chemical pollutants, including antibiotics, which are disposed of from households, hospitals, and factories. The WWTP aims to remove contaminants before disposing water in natural environments, e.g., streams, rivers, and lakes. While the reuse and reclamation of water through WWTPs reduce water shortage, they assist in the spread and emergence of AMR (Michael et al., 2013). This impacts the economy and society of countries affected by AMR (Bürgmann et al., 2018; Prüss-Ustün et al., 2019). It could be possible that proper sanitation and safe drinking water in the high-, middle-and low-income countries affect the emergence and spread of AMR. Although the removal of contaminants is successful, some ARB are not removed by the WWTP and are released with the effluent, which can spread to other natural environments (Meena et al., 2015).

Since WWTPs receive a diverse range of ARB and their genes from different sources. This ARB accumulates in the WWTP, and these high concentrations provide a selective pressure that facilitates the emergence of new ARB strains (Bürgmann et al., 2018). This is seen in wastewater treatment that uses biological treatment processes such as activated sludge, creating a favourable environment for ARB, ARGs, and their transmission (Guo et al., 2017). Contradictorily, it was hypothesized that the WWTP is efficient in removing ARBs which was seen in a study of the Dutch (Pallares-Vega et al., 2019). It could be argued that AMR in WWTP decreased since the Netherlands decreased antibiotic use as a whole. While this can be observed, antibiotic residues are not fully removed from the WWTP and persist in lower concentration when released with the effluent which serves as a selection pressure for ARBs (Keen and Fugère, 2017). WWTPs should revise their treatment options to manage AMR concerns while also providing sanitary water to communities. Policies about sludge and biosolid disposals are needed along with better infrastructure which will help decrease the spread of AMR to other sectors (Bürgmann et al., 2018; Kraemer et al., 2019).

#### Blue/green water

Freshwater environments are among the natural environments susceptible to contamination with antibiotics. These are released through different sources such as agricultural run-off, sewage discharge, and leaching from farms (Hutton et al., 2007; Hutton and Chase, 2017). The combination of antibiotics with a high density of bacteria provides a favourable environment for the development of ARGs (Marti et al., 2014). ARB carrying resistance genes can persist in freshwater and ultimately return to humans and animals by horizontal gene transfer (HGT; Chen et al., 2017; Mirza et al., 2020). Freshwater serves as a source of drinking water, recreational purposes and for agricultural practices, which use 70% of all freshwater, e.g., aquifers, streams, and lakes (Bürgmann et al., 2018). Continuous use of freshwater encourages the spread of AMR and the emergence of new pathogens, which leads to an increased risk of infection and prolonged and untreatable infections in humans, animals, and the environment (Nnadozie and Odume, 2019).

To date (Table 5), there is evidence showing that the consumption of contaminated water poses health risks (Jjemba et al., 2010; Shi et al., 2013; Sanganyado and Gwenzi, 2019) and that the WWTP has the potential to contribute to the dissemination of AMR in receiving rivers (Tang et al., 2016; Guo et al., 2017). Unfortunately, information on antibiotics in streams, lakes, beaches, pond water, surface and groundwater, and drinking water is scarce as it is presumed that antibiotic concentrations are naturally low in these areas. However, low antibiotic concentrations still select for ARB (Nnadozie and Odume, 2019). Studies also focus on ARB and genes at discharge points, i.e., receiving river or effluent, of the WWTP. At this point, the concentration of AMR is high but declines further away from the WWTP, downstream, which could be a result of degradation through biological or non-biological processes, uptake by aquatic microorganisms through HGT, diluted, absorption onto particulate matter, or transportation into different water systems (Anyaduba, 2016; Mirza et al., 2020). More focus should be on AMR within each freshwater environment, its link to different sectors, AMR after the WWTP discharge points and in potable water to gain a more holistic view (Mirza et al., 2020).

### Aquaculture

Aquaculture refers to the breeding, rearing, and harvesting of aquatic organisms in different water environments. This term is commonly used to describe fish farming. Aquaculture contributes to more than half of the world’s seafood consumption and production and has increased by 6% a year since 2001 (Rocha et al., 2022). The aquacultural sector is a complex interconnected system influenced by the environment, cultural, human, and economic factors (Brunton et al., 2019; Schar et al., 2021).

Like livestock production, antibiotics are used in aquaculture as a therapeutic agent to prevent and treat bacterial outbreaks. High concentrations are used due to high stocking densities, and the lack of individual treatment (Yukgehnaish et al., 2020).

Antibiotics are mixed with feed before being administered to animals or can be directly applied to the aquatic environment. This leads to the dispersal and leaching of antibiotics into the environment (Brunton et al., 2019). The gut microbiota of fish is also affected by the continuous use of antibiotics which alter the benefit of host-microbiota relationships allowing the gut microbiota to become resistant to antibiotics that are administered (Tyagi et al., 2019). Aquaculture is also affected by toxic materials such as silver and mercury, which co-resist and co-regulate with antibiotics. These toxic materials accumulate in fish bodies and the aquatic food web. This toxicity can be passed to humans and animals through consumption. It can affect the nervous system leading to death, as discussed by (Sonone et al., 2020), who reviewed the various heavy metal sources, i.e., agricultural activities, electronic waste, mining, industrial effluents, power plants, and biomedical waste and its role in degrading the aquaculture population, causing physical deformities in organisms and polluting the aquatic environment.

Several studies (Table 5) have shown that the excess use of antibiotics has led to the emergence of AMR. It cannot be ignored that the aquaculture industry is integrated with sewage, industrial wastewater, and land agriculture as manure and other agricultural residues are used in fish food. Areas in Western Europe and North America have banned antibiotics as a growth promoter, but it is still used as a therapeutic agent in fish food. Since 70 to 80% of antibiotics given to fish are excreted into the water, the entire body of water is exposed. This leads to leaching into different water environments and sediments, which provide long-term selection pressure in the aquatic environment, favoring horizontal gene transfer and the spread of AMR, as discussed by (Taylor et al., 2011; Watts et al., 2017; Mirza et al., 2020). Such policies on aquacultural practices should be reviewed to decrease the incidence of ARB and infections (Marti et al., 2014; Pérez-Sánchez et al., 2018; Preena et al., 2020a; Zhao et al., 2020).

### Soil

Microbes in soil are of great importance as they break down organic matter, recycle nutrients, bioremediation, and produce antibiotics (Chandra and Kumar, 2017). Variation in biotic, e.g., plants, animals and other bacteria, and abiotic, e.g., water, soil and atmosphere, conditions of soil cause the residing microbes to adapt and develop strategies for survival and successful reproduction. Antibiotic production is the most powerful adaptation strategy from soil microbes to inhibit the growth of competing microbes (Chandra and Kumar, 2017). This strategy has led to the natural development of AMR. Antibiotics and disinfectants used in medicine, agriculture, and aquaculture have been the driver of AMR through manure/fertiliser application on soil, irrigation, and run-off. Consequences of these are the emergence of new ARGs and the spread of pathogenic ARB to other environments, humans, and animals (Martínez, 2008; Wang et al., 2015; Chandra and Kumar, 2017).

Although AMR research has shown that WWTP effluents can still contain a high level of ARGs and bacteria after disinfection, there is not much research on the sludge and biosolids applied to soil and how this changes the bacterial community and resistome (Anyaduba, 2016; Nnadozie and Odume, 2019). The application of manure/fertiliser/sludge to soil should also be looked into to determine if there is any difference in soil and crop quality as they are used in agricultural and remediation practices. Studies have shown the prevalence of AMR in soil and how bioremediation and pollutants have facilitated its spread (Table 5).

### Plants

The agricultural industry has been valued at an estimated US$ 3.2 trillion worldwide and accounts for the largest share of the GDP and employment in developing countries and underdeveloped nations. Unfortunately, the agriculture industry has been suffering due to population growth, pest resistance, and the burden of natural resources (Raman, 2017).

Manure is commonly used as fertilizer on vegetable farmlands to enhance crop yield. This enhances AMR as 30–90% of antibiotics consumed by animals are excreted in faeces which is used as manure. Using sewage sludge and water for irrigation contributes to rising AMR in agriculture (Minden et al., 2017; Grenni et al., 2018; Spielmeyer, 2018). Crops can take up antibiotics through water transport and passive absorption (Hu et al., 2010). Antibiotic-resistant bacteria found in soil can reach the interior of fruits and vegetables either through germination seeds in contaminated soil or the direct transmission of bacteria from the soil to the plant *via* irrigation. Both these routes can cause the bacteria to colonize in the roots and the edible parts of the plant (Hirniesen et al., 2012; Alegbeleye et al., 2018; Sundin and Wang, 2018).

These edible plants are consumed by animals or sold as ready-to-eat products such as bagged salads or pre-cut vegetables, which may enhance the proliferation and survival of ARB (Jung et al., 2014; Rajwar et al., 2016; Wadamori et al., 2017; Mir et al., 2018). This is due to many factors such as hygiene standards, irrigation water, temperature, storage, pH, soil, manure, and antimicrobial agents. Since these products rarely go through a heating step before consumption, it increases the risk of consumers being exposed to high numbers of different pathogenic ARB (Mir et al., 2018). This poses a threat to society as consumers who opt for ready-to-eat products are faced with the risk of acquiring foodborne illnesses which could be untreatable with antibiotics. Table 5 summarizes studies showing that manure, fertilizers, compost, and irrigation are all routes for the transmission and dissemination of AMR to plants.

## Antibiotic resistance and its transmission

The use and misuse of antibiotics in humans, animals, and the environment have been linked to the emergence of ARB in each of these sectors. Antibiotic residues, ARB, and ARGs, are spread to different environments (Miłobedzka et al., 2021). Most antibiotics are excreted in animal urine and faeces (Jayalakshmi et al., 2017). This is introduced to the environment directly by manure amendment, which provides nutrients for crops, or through water which is the primary link between humans, animals, and nature (Miłobedzka et al., 2021).

Since antibiotics are used in the food animal industry, ARB can spread *via* run-off, which can go into lakes, streams, and rivers. Water from these areas is used for irrigation for agricultural practices. This leads to the spread of ARB from food animals to water, soil, and crops (Figure 1). This interconnectedness facilitates the exchange of AMR and allows for the emergence of new genetic determinants. This emergence and spread can occur by HGT (Schmieder and Edwards, 2012; Pan and Chu, 2017; Sarkar et al., 2018; Mahmood et al., 2019).

### Horizontal gene transfer

ARB exists across animal, human and environmental sectors. The evolution of AMR and the dynamics of ARGs spread across these sectors are critical for predicting emerging pathogens and controlling AMR dissemination. HGT is primarily responsible for rapid AMR spread and dissemination of ARGs between bacteria and across species (Sun et al., 2020; Vinayamohan et al., 2022). AMR can be established either vertically by point mutations or horizontally by acquiring MGEs such as plasmids and transposons (Vinayamohan et al., 2022).

HGT is a process that introduces variation in the bacterial genome under natural selection. It is an essential process that allows bacteria to adapt to environmental changes such as exposure to antibiotics (Hall et al., 2020).

HGT predates the production and use of antibiotics. Unfortunately, the increased usage of antibiotics has put selective pressure on bacterial strains. This has sped up the process of bacteria becoming antibiotic-resistant by various genotypic and phenotypic resistance mechanisms. This diversification can be seen in the resistance to antibiotics of different classes (Power, 2019).

Genes that confer resistance can either be intrinsic or extrinsic (Ali et al., 2018). Intrinsic resistance is the bacterium’s innate ability to resist the antimicrobial agent’s activity. This occurs through structural or functional characteristics. Extrinsic or acquired resistance occurs when a previously susceptible bacterium obtains the ability to resist the activity of the antimicrobial agent. This can result from the mutation of genes involved in normal physiological processes, the acquisition of foreign resistance genes, or a combination of both (Blair et al., 2015; von Wintersdorff et al., 2016; Kumar, 2017).

Pathogenic bacteria can acquire resistance *via* HGT. This has caused AMR to spread from commensal and environmental bacteria to pathogenic ones. ARGs are often carried in MGEs, e.g., plasmids, transposons, or integrons which act as vectors for transferring genetic information between bacterial cells. The three classical pathways of HGT are conjugation, transformation, and transduction. These HGT mechanisms lead to increased bacteria fitness, which is essential for survival in the presence of antibiotics (Capita and Alonso-Calleja, 2013; Munita and Arias, 2016; von Wintersdorff et al., 2016; Kumar, 2017; Vinayamohan et al., 2022).

Recent studies have identified other mechanisms by which DNA can transfer between hosts. Gene transfer agents are DNA-containing particles, similar to phages, but cannot carry genes for particle production (Redfield and Soucy, 2018). Genes can also be transferred between bacteria that form intercellular connections by nanotubes or membrane fusion (Hall et al., 2017, 2020). Some bacteria can also release DNA-containing membrane-bound vesicles that carry genetic information to new hosts. Interestingly, some of these mechanisms are not under bacterial control but are controlled by semi-autonomous segments of DNA (Hall et al., 2017).

Bioinformatic approaches such as MetaCHIP (Song et al., 2019) and WAAFLE (WAAFLE, 2022) can be used to detect HGT events in metagenomic sequences. These processes involve an all-against-all BLASTN or BLASTX of genes within assembled contigs, and potential HGT events are determined based on genes with the best hits in other taxonomic groups (Douglas and Langille, 2019). These approaches are limited by sequencing technologies and assemblers, which often fail to assemble long regions with high sequence similarity (Song et al., 2019). It can be assumed that inferring HGT events will become easier as the assembly of metagenomic sequences improves (Douglas and Langille, 2019).

### Mobile genetic elements

Specialized MGEs mediate HGT between bacteria. These play an important role in bacterial ecology and evolution. Several factors impact HGT in bacteria, such as gene expression, protein connectivity, and biochemical properties; however, the range of genes carried on MGEs remains unknown (Rodríguez-Beltrán et al., 2020).

MGEs are segments of DNA that encode enzymes and other proteins that mediate the movement of DNA within or between bacterial cells (Hall et al., 2017; Emamalipour et al., 2020). MGEs carry genes other than those necessary for transfer and replication (Hall et al., 2017). These accessory genes do not play a role in their vertical or horizontal transmission but affect the success of MGEs. The range of accessory genes encoded by MGEs and their ability to be phenotypically expressed in different genetic backgrounds are critical in its evolution (Rodríguez-Beltrán et al., 2020).

The most important elements that play a role in HGT are the conjugative and mobilized elements. Mobilized elements contain all genetic information required to transfer from one bacterium to another, while conjugative elements use conjugation functions of elements such as plasmids and transposons to transfer from one host to another. These MGEs can carry multi-drug resistant (MDR) plasmids and give rise to MDR bacteria *via* HGT (Vinayamohan et al., 2022). Bacteriophages also play a role in the spread of DNA by the transduction process. Some elements can translocate to new sites on a genome but cannot transfer to a new bacterial host. These include the transposons and mobile integrons (Van Hoek et al., 2011; Hall et al., 2017).

### Detecting mobile genetic elements

The ability of MGEs containing ARGs to spread is controlled by the genetic elements and host factors. The genetic elements and host factors control the ability of MGEs containing ARG, but most important is the selective pressure in the environment. When antibiotics are present in the environment, there is strong selective pressure on the spread of resistance. Elements that promote resistance will be selected for, and those stopping the spread of mobile elements will be selected against (von Wintersdorff et al., 2016; Hall et al., 2017; Partridge et al., 2018). To control the spread of AMR, it is important to understand MGEs and the ecology of the environments in which they spread easily (Van Hoek et al., 2011; Slizovskiy et al., 2020).

Several databases such as ACLAME (Leplae et al., 2004, 2010), ISFinder (Siguier et al., 2006), INTEGRALL (Moura et al., 2009), and ICEberg (Liu et al., 2019) and web-based tools such as ISSaga2 (Varani et al., 2011), MobilomeFinder (Ou et al., 2007), oriTfinder (Li et al., 2018), TnpPred (Riadi et al., 2012) and MobileElementFinder (Johansson et al., 2021) were developed to identify MGEs in metagenomic datasets (Peng et al., 2021). These web-based tools and databases range from detecting plasmids, integrons, transposons, prophages, and insertion sequences.

Overall, there is no single solution for detecting MGEs as these databases, and web-based tools are incomplete and biased towards pathogens studied extensively. This makes it difficult and time-consuming as most web-based tools and databases need to be evaluated first and used in combination to provide an all-inclusive view on MGEs associated with AMR (Rankin et al., 2011; Brito, 2021). This information can be applied to clinical and non-clinical sectors to potentially influence policies and strategies which can help control the spread of AMR (Sun et al., 2020).

## Outlook

Antibiotic resistance affects animals, plants, the environment and human health (Malagón-Rojas and Lagos, 2020). Monitoring and understanding the prevalence, mechanisms and spread of AMR is a priority in non-clinical sectors (Boolchandani et al., 2019). Since measures against AMR are focused on the clinical health sector, the environment is under-represented which allows for significant gaps in data, research and control strategies therefore failing to link the effects of AMR from one sector to the other (Malagón-Rojas and Lagos, 2020).

Metagenomic sequencing allows for the detection of known and novel ARGs, HGT events, and associated MGEs in complex communities providing in-depth information on their prevalence, distribution, and transmission in non-clinical sectors such as the animal industry, agriculture, and the environment (Forbes et al., 2017).

While metagenomics is promising, its implementation in non-clinical sectors is still in its early stages. Metagenomic sequencing creates a vast amount of data and requires specialized bioinformatic expertise, which makes this a costly approach (Forbes et al., 2017; Oniciuc et al., 2018). To date, only a few countries and laboratories have the resources and expertise to use metagenomic approaches as a surveillance system for AMR. In addition, bioinformatics methodologies need to be standardized and constantly updated and curated to allow for an accurate comparison between various samples, as the selection of bioinformatic tools and AMR databases does have a significant impact on the results (Hendriksen et al., 2019; Lal Gupta et al., 2020).

Nevertheless, using bioinformatic tools and databases can assist in identifying risky practices, effects of antibiotic usage, and hotspots of AMR. This can facilitate the design of new policies to control the spread of AMR between clinical and non-clinical sectors (Shen and Zhao, 2018; Nijsingh et al., 2019). Such knowledge and practices are urgent as these sectors, including the public health sector, are faced with a future of untreatable infections, increasingly costly medicinal treatment, a higher cost of living, and an increased mortality rate (Bengtsson and Greko, 2014; Littmann et al., 2015; Nijsingh et al., 2019).

## Conclusion

The application of metagenomic and bioinformatic approaches to AMR research can provide fast and reliable predictions of AMR and antibiotic use in various non-clinical sectors. These hold a great promise for understanding AMR molecularly, predicting outbreaks and transmission, and emerging pathogens. This information can lead to better policymaking in each sector and decrease the incidence of infections as conditions for the animal industry, agriculture, and the environmental sectors are improved.

## Author contributions

SP and TA conceived, designed and wrote the manuscript. AU and DC-F edited and proof-read the manuscript. All authors contributed to the article and approved the submitted version.

## Funding

This work is based on the research supported by wholly/in part by the National Research Foundation of South Africa (Grant Numbers: 120192).

## Conflict of interest

The authors declare that the research was conducted in the absence of any commercial or financial relationships that could be construed as a potential conflict of interest.

## Publisher’s note

All claims expressed in this article are solely those of the authors and do not necessarily represent those of their affiliated organizations, or those of the publisher, the editors and the reviewers. Any product that may be evaluated in this article, or claim that may be made by its manufacturer, is not guaranteed or endorsed by the publisher.
